# Polyamine Metabolites Profiling for Characterization of Lung and Liver Cancer Using an LC-Tandem MS Method with Multiple Statistical Data Mining Strategies: Discovering Potential Cancer Biomarkers in Human Plasma and Urine

**DOI:** 10.3390/molecules21081040

**Published:** 2016-08-10

**Authors:** Huarong Xu, Ran Liu, Bosai He, Cathy Wenchuan Bi, Kaishun Bi, Qing Li

**Affiliations:** 1 School of Pharmacy, Shenyang Pharmaceutical University, 103 Wenhua Road, Shenyang 110016, China; huarongxu@outlook.com (H.X.); liuran8515@hotmail.com (R.L.); jesse83@163.com (B.H.); kaishunbi.syphu@gmail.com (K.B.); 2 Division of Life Science and Center for Chinese Medicine, The Hong Kong University of Science and Technology, Clear Water Bay, Kowloon, Hong Kong, China; bccbb@ust.hk

**Keywords:** plasma and urine polyamine metabolites, lung and liver cancer characterization, UHPLC-MS/MS, statistical data mining, cancer biomarker

## Abstract

Polyamines, one of the most important kind of biomarkers in cancer research, were investigated in order to characterize different cancer types. An integrative approach which combined ultra-high performance liquid chromatography—tandem mass spectrometry detection and multiple statistical data processing strategies including outlier elimination, binary logistic regression analysis and cluster analysis had been developed to discover the characteristic biomarkers of lung and liver cancer. The concentrations of 14 polyamine metabolites in biosamples from lung (*n* = 50) and liver cancer patients (*n* = 50) were detected by a validated UHPLC-MS/MS method. Then the concentrations were converted into independent variables to characterize patients of lung and liver cancer by binary logic regression analysis. Significant independent variables were regarded as the potential biomarkers. Cluster analysis was engaged for further verifying. As a result, two values was discovered to identify lung and liver cancer, which were the product of the plasma concentration of putrescine and spermidine; and the ratio of the urine concentration of *S*-adenosyl-l-methionine and *N*-acetylspermidine. Results indicated that the established advanced method could be successfully applied to characterize lung and liver cancer, and may also enable a new way of discovering cancer biomarkers and characterizing other types of cancer.

## 1. Introduction

According to an American Cancer Society report in 2013, lung cancer is the most common cancer in terms of morbidity and mortality among both men and women. It has been estimated that over 1.6 million new cases and 1.4 million deaths of lung cancers have occurred worldwide [1]. As a common malignant tumor, lung cancer also seriously threatens human health and life in China as well. Liver cancer is one of the most common cancers around the world, and its morbidity is ever-increasing as a consequence of viral hepatitis, long-term alcohol consumption and unhealthy lifestyles. The situation in China is even worse. Of all the new liver cancer cases around the world, China accounts for 55 percent, and the incidence of the liver cancer in China has been continuously increasing over the years. Consequently, both lung cancer and liver cancer have become the common and frequently-occurring diseases that threaten human health. Nowadays the common cancer treatments not only depend on effective individualized treatments, but also on early diagnosis and prevention. For a better solution to these common cancers, effective individualized treatments are not enough; early detection and prevention are also highly required. Thus, the diagnosis, monitoring and characterization of lung and liver cancer are important cancer research missions. The analysis of metabolite biomarkers now provides an ideal approach for generating novel, non-invasive diagnostic tests in disease research.

**Figure 1:**
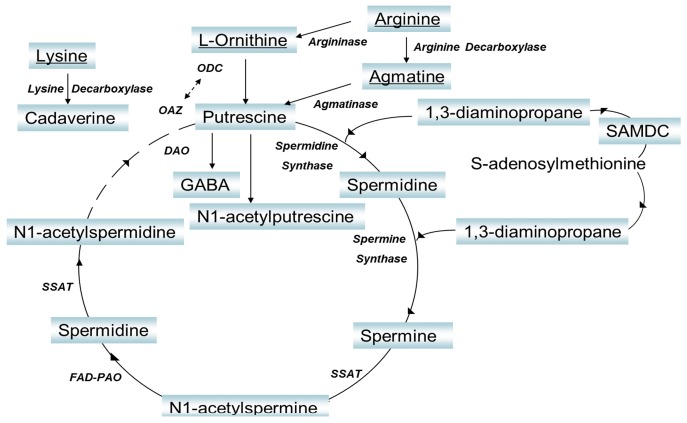
The anabolic biosynthetic and catabolic pathways of polyamines metabolome, where 1,3-diaminopropane, putrescine, cadaverine, spermidine, spermine, agmatine s, *N*-acetylputrescine, *N*-acetylspermine, *N*-acetylspermidine are polyamines, l-ornithine, lysine, l-arginine, *S*-adenosyl-l-methionine are amino acid which can form polyamines, and γ-aminobutyric acid are catabolic of polyamines. The notations here are ornithine decarboxylase (ODC), diamine oxidase (DAO), *S*-adenosylmethionine decarboxylase (SAMDC), Spermidine and spermine acetyl transferase (SSAT) and flavin adenine dinucleotide-polyamine oxidase (FAD-PAO).

Polyamines are one of the most important kind of biomarkers in cancer research. During rapid tumor growth periods, the metabolism of polyamines is disturbed and their concentration is elevated [2,3,4,5,6]. Polyamine levels in vivo can be recognized as a dynamic equilibrium between their anabolism and catabolism, as shown in Figure 1.

This equilibrium might be disrupted during cancer evolution. Meanwhile, different levels of polyamines in body fluids caused by different cancers may result from the fact that different cancers have different metabolisms. Consequently, the detection of the fluctuating concentrations of polyamines in body fluids may help diagnose cancer or characterize different cancer types. There are considerable reported references studying the levels of polyamines to reveal the differences between cancer and benign samples [7,8,9,10], but the polyamine metabolite profile has not been deeply and systematically explored. Currently, the application of polyamines in early diagnosis of cancer is still limited, especially in characterizing different type of cancers. Studies on available biomarkers for specific cancers are of great significant in cancer diagnosis. Moreover, researching the targeted metabolic pathway would be the key for cancer discrimination. In our previous study, it has been confirmed that the levels of polyamines in cancerous human plasma and urine were significantly higher than those in healthy volunteers [7,8,11]. The current study was undertaken to elucidate the role of polyamines as biomarkers for cancer discrimination. In particular, we focused on the concentrations of polyamines in metabolite profiles to evaluate the possible differences between lung and liver cancer. Development of a discrimination method for lung and liver cancer was also explored. Our previous study had developed an ultra-high performance liquid chromatography-tandem mass spectrometry (UHPLC-MS/MS) method to determine fourteen analytes from polyamine metabolite profiles and described the changes in plasma and urine of both liver cancer patients and healthy volunteers [11]. In this study, polyamine metabolite profiles in plasma and urine from 50 liver and 50 lung cancer patients were analyzed. A Hampel’s test was firstly adopted to remove the high through determination error-derived outliers, which may affect the precision of the method. The values of fourteen analytes could then be converted into independent variables for distinguishing between patients with lung and liver cancer by logic regression analysis. Finally, a cluster analysis method was adopted to verify the discrimination of the two types of cancer. This classification method might be a useful tool in diagnosis and prediction of different cancer types. Also, it could expand the application range of polyamines in cancer research.

## 2. Results and Discussion

### 2.1. Levels of Plasma and Urine Polyamines in Lung and Liver Cancer Patients

The concentrations of fourteen analytes in polyamine metabolite profiles in lung and liver cancer patients, including 1,3-diaminopropane, putrescine, cadaverine, spermidine, spermine, agmatine, *N*-acetylputrescine, *N*-acetylspermine, *N*-acetylspermidine, l-ornithine, lysine, l-arginine, *S*-adenosyl-l-methionine, γ-aminobutyric acid, were determined by UHPLC-MS/MS detection. Table 1 summarizes the concentrations (mean ± SD) of the fourteen analytes in plasma and urine of 50 liver and 50 lung cancer patients. The results revealed that in liver cancerous plasma, the most abundant polyamine was putrescine, followed by spermidine, spermine, *N*-acetylspermine and *N*-acetylspermidine in decreasing order. Meanwhile in lung cancerous plasma, the most abundant polyamine was putrescine, followed in decreasing order by spermine, spermidine, *N*-acetylspermidine and *N*-acetylspermine, while in liver cancerous urine, the most abundant polyamine was spermine, followed by *N*-acetylspermidine, putrescine, cadaverine, spermidine, *N*-acetylspermine and *N*-acetylputrescine in decreasing order. In lung cancerous urine, the most abundant polyamine was *N*-acetylspermidine, followed by spermine, putrescine, cadaverine, spermidine, *N*-acetylspermine, and *N*-acetylputrescine in decreasing order. Since polyamine levels differed in the two cancer types, a discrimination method was developed to classify lung and liver cancer. The amounts of polyamine metabolome in plasma and urine from 50 lung, 50 liver cancer patients and heathy volunteers were shown in Tables S1 and S2.

### 2.2. Classify the Lung and Liver Cancer Using Plasma and Urine Polyamine

**Figure 2:**
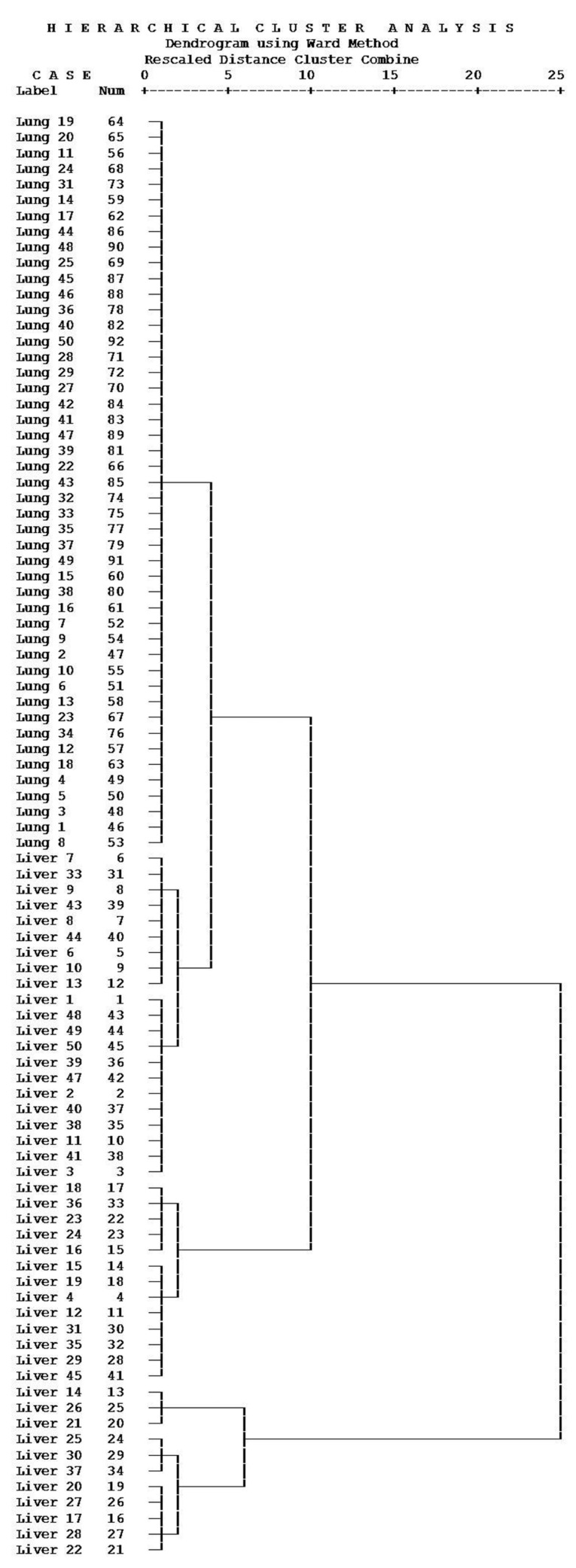
Result of the cluster analysis for liver and lung cancer plasma, the Ward’s method was adopted and the Chebychev method was chosen as measurement.

Prior to all in-depth data analysis, data quality was assessed for outlier removal. The raw data from chromatographic analysis of samples was processed by Hampel’s test, which is efficient in detecting outliers, no matter how many outliers are present in a sample [12,13]. One hundred raw data from cancerous plasma samples underwent outlier removal with Hampel’s test. Data from five liver and three lung cancer plasma was thus removed. In the following logic regression analysis, using the conditional method, liver cancer plasma and lung cancer plasma were assigned as 0 and 1, respectively, and used as dependents, while the fourteen analytes were adopted as covariates. As displayed in Table 2 and Table 3, we got putrescine (x_1_) and spermidine (x_2_) as two variables in the regression equation *y* = 9.424 – 0.112*x*_1_ − 0.304*x*_2_. The correlation value of the two characteristic variables was 0.859, which indicated a positive correlation between putrescine and spermidine. Hence, we took the product of putrescine and spermidine as argument to characterize liver and lung cancerous plasma. Then Ward’s method and Chebychev measurement was adopted to evaluate the similarity of samples. As displayed in Figure 2, 92 plasma samples were gathered into two groups, the liver cancer samples were classified as Group I and the lung cancer samples were classified as Group II. The results showed a significant difference between liver and lung cancer samples using plasma putrescine and spermidine as indicators.

**Figure 3:**
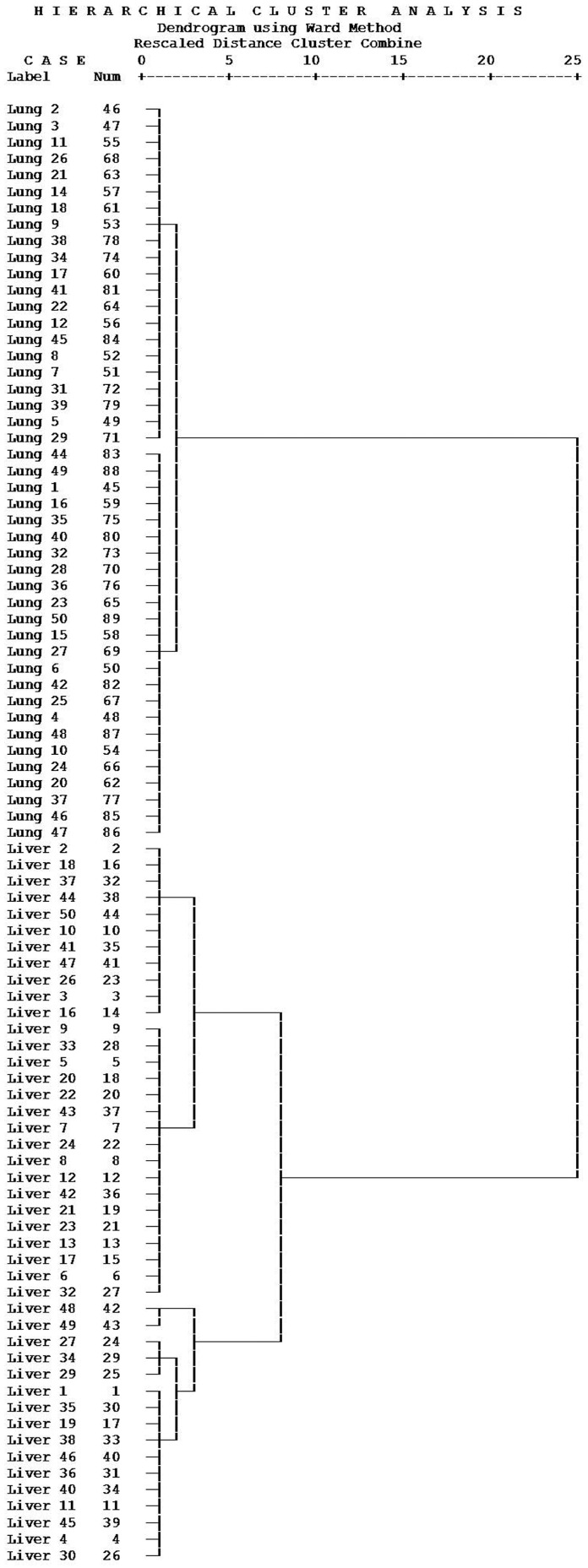
Result of the cluster analysis for liver and lung cancer urine, the Ward’s method was adopted and the Chebychev method was chosen as measurement.

Similarly, one hundred raw data from urine samples underwent the same analysis as the plasma samples, and six sets of liver and five of lung cancer urine data were removed by the Hampel’s test. As displayed in Table 4 and Table 5, we got *S*-adenosyl-l-methionine (x_1_) and *N*-acetylspermidine (x_2_) as two variables in the regression equation *y* = −0.395 + 0.001*x*_1_ − 0.03*x*_2_. Their correlation coefficient was −0.905, indicating a negative correlation between *S*-adenosyl-l-methionine and *N*-acetylspermidine. Therefore, we took the ratio of *S*-adenosyl-l-methionine and *N*-acetylspermidine as argument to characterize liver and lung cancerous urine. As displayed in Figure 3, 89 urine samples were gathered into two groups, where the liver cancer samples were classified as Group I and the lung cancer samples were classified as Group II. These results revealed a significant difference between liver and lung cancer samples when using urine *S*-adenosyl-l-methionine and *N*-acetylspermidine as indicators.

The levels of plasma putrescine and spermidine in liver cancer patients were considerably higher than those in lung cancer patients. Moreover, the product of the concentrations of putrescine and spermidine in liver cancer patients was at least forty times that in lung cancer patients. Considering the interconnected biosynthetic and catabolic pathways of the polyamine metabolome described in Figure 1, putrescine was derived from l-ornithine by ornithine decarboxylase (ODC) catalysis. Putrescine was converted into spermidine by spermidine synthetase catalysis, and spermidine was further transformed into spermine by spermine synthetase. It can be inferred that the major differences between liver and lung cancer cell proliferation in blood circulation were a remarkable increase of ODC and spermidine synthetase activity, and a decrease of spermine synthetase activity. In addition, the pathway from putrescine to spermine could be promoted during liver cancer cell proliferation. As a result, it is reasonable to use the product of putrescine and spermidine concentration as a diagnostic marker to characterize liver and lung cancer. Further research is required to study the main reasons affecting the activity changes of ODC, spermidine synthetase and spermine synthetase. Likewise, the levels of *S*-adenosyl-l-methionine in liver cancer patient urine were considerably higher than in lung cancer patients, while the levels of *N*-acetylspermidine in liver cancer patient urine were considerably lower than in lung cancer patients. Hence the ratio of *S*-adenosyl-l-methionine and *N*-acetylspermidine in urine from liver cancer patients was markedly higher than that from lung cancer patients. In the polyamine metabolic process, *S*-adenosyl-l-methionine is derived into 1,3-diaminopropane and further forms spermine, which is the precursor of *N*-acetylspermidine. It is suggested that during the blood clearance and metabolism process, the pathway from *S*-adenosyl-l-methionine to *N*-acetylspermidine was restrained during the liver cancer cell proliferation, while spermine synthetase, flavin adenine dinucleotide–polyamine oxidase (FAD-PAO) and spermidine/spermine *N*-acetyltransferase (SSAT) manifested lower activity in the cell proliferation of liver cancer than lung cancer. Thus further studies should be done on the influence of the decreasing activity of spermine synthetase, FAD-PAO and SSAT.

## 3. Materials and Methods

### 3.1. Materials

The reference standards of 1,3-diaminopropane, putrescine, cadaverine hydrochloride, spermidine hydrochloride, spermine, agmatine sulfate salt, *N*-acetylputrescine hydrochloride, *N*-acetylspermine trihydrochloride, *N*-acetylspermidine dihydrochloride, l-ornithine hydrochloride, lysine, l-arginine, *S*-adenosyl-l-methionine, γ-aminobutyric acid and 1, 6-diaminohexane (used as an internal standard) were all obtained from Sigma-Aldrich (St. Louis, MO, USA). HPLC grade methanol was purchased from Fisher Chemicals (Fair Lawn, NJ, USA). Heptafluorobutyric acid (HFBA) was obtained from Sigma-Aldrich. All the other reagents were of analytic grade. Redistilled and deionized water was used throughout the study.

### 3.2. Sample Collection

The study protocol and procedure were approved by the Ethics Committee of The General Hospital of Shenyang Military Region (Ref: 2012/7) and was carried out in accordance with the Declaration of Helsinki. All participants of this study gave written informed consent. Fifty healthy volunteers, 50 confirmed lung cancer only patients and 50 confirmed liver cancer only patients without any other diseases and complications were recruited. Cancer diagnoses were made on the basis of usual clinical and laboratory results and confirmed by tissue biopsies. The ratios of sexes were almost equal in the two groups. The average ages were 57 (age range from 45 to 77 years) among lung cancer patients and 53 years (age range from 27 to 77 years) among liver cancer patients. Plasma and urine samples were collected in polyethylene bottles and kept frozen at −80 °C until use.

### 3.3. UHPLC-MS/MS Analysis

Liquid chromatography was performed on a Prominence™ LC-20A UFLC XR system equipped with a binary pump, a degasser, an autosampler and a thermostatted column compartment (Shimadzu, Kyoto, Japan). A SHIM-PACK XR-ODS column (75 mm × 3.0 mm, 2.2 μm) (Shimadzu) protected by a high pressure column pre-filter (2 μm) were held at 30 °C. Chromatographic separation was achieved with gradient elution using a mobile phase composed of 0.05% HFBA in water (A) and 0.05% HFBA in methanol (B). The 9.0 min LC gradient program was as follows: 20% B→ 20% B for 0.01–2.00 min; 20% B→ 50% B for 2.01–4.00 min; 50% B for 4.01–6.00 min; 20% B for 6.01–9.00 min. Efficient and symmetrical peaks were obtained at a flow rate of 0.4 mL·min^−1^ with a sample injection volume of 5 μL.

The MS detection was carried out on a Sciex 4000 QTRAP MS/MS triple quadrupole – linear ion trap mass spectrometer equipped with a Turbo Ion Spray source (Sciex, Redwood City, CA, USA). All the operations, including data acquiring and analysis were controlled by Analyst software (version 1.5.2, Sciex). The analytes were detected in multiple reaction monitoring mode (MRM). An electrospray positive ionization (ESI+) was occupied with nitrogen as gas 1, gas 2, and curtain gas set at 50, 40, and 20 psi, respectively. The source temperature and Ion Spray Voltage were set at 500 °C and 5500 V. Declustering potential (DP), entrance potential (EP), collision energy (CE) and cell exit potential (CXP) values were optimized for each analytes in MRM measurements. The quantitative parameters were set according to our previously paper [11].

### 3.4. Sample Preparation

Samples were prepared as described in our previously paper [11]. Briefly, an aliquot of 50 μL IS (100 ng·mL^−1^) and 250 μL plasma or urine sample was transferred to an Eppendorf microtube, vortex-mixed with 50 μL methanol–water (20:80, *v/v*) for 30 s, followed by protein precipitation by adding 250 μL methanol (containing 0.1 % acetic acid), mixing for 5 min and centrifuging for 3 min at 15,000× *g* and 4 °C. The supernatant was transferred to another Eppendorf microtube and evaporated to dryness at 30 °C under air stream. The residue was dissolved with initial LC mobile phase and 5 μL of it was injected for analysis.

### 3.5. Method Validation

This method was validated according to the US FDA document and other related guidelines with respect to the limit of quantification, linearity, precision, accuracy, absolute recovery, matrix effect, dilution integrity and stability [14,15]. Samples were extracted as described in sample preparation and analyzed by UHPLC-MS/MS. Calibration standards and QC samples were prepared by adding a dilution of the stock analyte solution to blank plasma or urine samples on every validation day as described in our previous paper [11]. Polyamine concentrations were calculated from calibration curves while urinary polyamine concentrations were adjusted by the value of creatinine.

### 3.6. Data Analysis

**Figure 4:**
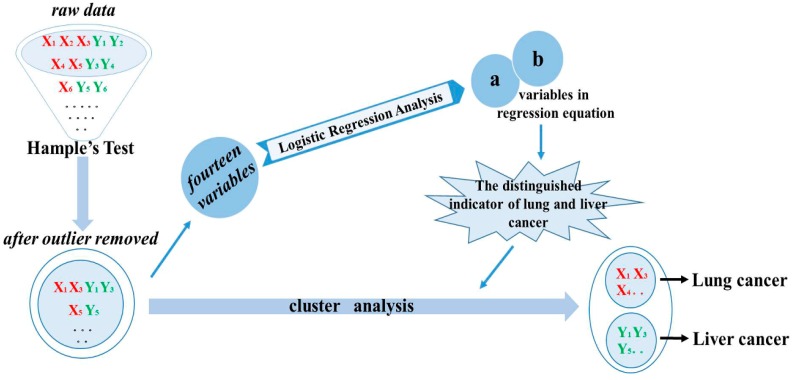
The visualization analysis process for the classifying lung and liver cancer.

As shown in Figure 4, the visualization analysis process for the predictive model was summarized. The raw data from chromatographic analysis of samples was processed by Hampel’s test with the Excel software (Office 2010, Microsoft Inc., Redmond, WA, USA). Hampel’s test procedure is as follows:

- Calculate the median x_m_ of all results x_i_, x_i_ ranging from x_1_ to x_n_.
- Calculate the absolute residuals r_i_ of single data from the median. r_i_ = ∣x_i_–x_m_∣
- Calculate the median r_m_ of absolute residuals.

Value was considered as an outlier when its absolute residual was 4.5 times larger than rm. After the removal of outliers, the lung and liver cancer which assigned as 0 and 1 respectively were used as dependents, while fourteen polyamine metabolome served as variables. These values were analyzed by logistic regression analysis with SPSS 19.0 (SPSS Inc., Chicago, IL, USA). Then the classical variables in logistic regression equation, whose significant level were less than 0.05, were considered as the factor in characterizing lung and liver cancer. According to the correlation coefficient between the two variables, the ratio or product of the variables was adopted for further analysis. Finally, by means of cluster analysis, we take the ratio or product as indicators to classify lung and liver cancer.

## 4. Conclusions

In the study, polyamines metabolites from cancer plasma and urine samples were investigated as biomarkers for the characterization of lung and liver cancer. The integrative method involved analyte concentration acquisition, outlier removal by Hampel’s test, logic regression analysis and cluster analysis. The results revealed the different metabolisms in the cell proliferation processes of liver and lung cancer, and that characteristic polyamines can assist in identifying different types of cancer. It can be concluded that the product of the plasma concentration of putrescine and spermidine and the ratio of the urine concentration of *S*-adenosyl-l-methionine and *N*-acetylspermidine can be used as discrimination markers for liver and lung cancer. The method may become a promising tool in diagnosing lung and liver cancer patients in cancer screening tests. The high throughput, high sensitivity metabolomics approach which combined UHPLC-MS/MS analysis with statistical methods showed excellent potential in studying cancer characterization. The approach could also be applied in many other aspects and provide useful scientific data.

## Figures and Tables

**Table 1:** Amounts of polyamine metabolome in plasma (ng/mL) and urine (ng/mg creatinine) from lung and liver cancer patients (mean ± SD).

Polyamine Metabolome	Plasma (ng/mL)		Urine (ng/mg Creatinine)	
	Lung Cancer Patients	Liver Cancer Patients	Lung Cancer Patients	Liver Cancer Patients
DAP ^a^	3.74 ± 2.26	5.79 ± 4.54	0.98 ± 0.88	1.42 ± 2.09
PUT ^a^	35.65 ± 16.41	77.11 ± 37.14 **	19.77 ± 9.13	35.12 ± 44.46 **
CAD ^a^	2.58 ± 2.03	3.09 ± 2.43	14.38 ± 16.25	26.33 ± 28.77
SPD ^a^	5.55 ± 6.79	46.24 ± 30.84 **	8.88 ± 7.87	6.85 ± 8.04
SPM ^a^	6.78 ± 3.87	14.24 ± 10.73 **	90.56 ± 97.67	212.46 ± 264.63
AGM ^a^	71.79 ± 21.97	67.08 ± 46.67	5544 ± 466	5396 ± 4099
l-ORN ^a^	6374 ± 2429	9.85 × 10^3^ ± 5.69 × 10^3^	202.1 ± 170.8	256.81 ± 305.2
LYS ^a^	1.440 × 10^5^ ± 0.613 × 10^5^	1.411 × 10^5^ ± 0.789 × 10^5^	2.844 × 10^4^ ± 2.537 × 10^4^	2.878 × 10^4^ ± 2.303 × 10^4^
l-ARG ^a^	1.042 × 10^5^ ± 0.468 × 10^5^	1.005 × 10^5^ ± 0.636 × 10^5^	257.7 ± 281.2	699.2 ± 549.6 **
SAM ^a^	159.7 ± 90.1	131.7 ± 129.0	2283 ± 2074	6880 ± 5074 *
*N*-PUT ^a^	0.36 ± 0.24	0.52 ± 0.49	1.00 ± 1.09	0.58 ± 0.80
*N*-SPM ^a^	3.12 ± 1.32	7.40 ± 3.39 **	4.44 ± 4.12	1.82 ± 1.74 *
*N*-SPD ^a^	3.15 ± 0.87	6.21 ± 3.78 **	300.1 ± 38.32	111.6 ± 128.3
GABA ^a^	74.13 ± 32.86	91.12 ± 70.48	16.07 ± 12.00	22.03 ± 16.57

* *p* < 0.05, compared to lung cancer patients. ** *p* < 0.01, compared to lung cancer patient; ^a^ DAP for 1,3-diaminopropane, PUT for putrescine, CAD for cadaverine, SPD for spermidine, SPM for spermine, AGM for agmatine, *N*-PUT for *N*-acetylputrescine, *N*-SPM for *N*-acetylspermine, *N*-SPD for *N*-acetylspermidine, l-ORN for l-ornithine, LYS for lysine, l-ARG for l-arginine, SAM for *S*-adenosyl-l-methionine and GABA for γ-aminobutyric acid.

**Table 2:** The variables in the equation of logic regression analysis for liver cancer plasma (assigned as 1 as dependent) and lung cancer plasma (assigned for 2 as dependent) using the Conditional Method as analytical method.

		B	S.E.	Wald	df	Sig.	Exp (B)
**Step 1 ^a^**	putrescine	−0.099	0.020	24.383	1	0.000	0.906
	Constant	3.622	0.702	26.647	1	0.000	37.414
**Step 2 ^b^**	putrescine	−0.112	0.036	9.478	1	0.002	0.894
	spermidine	−0.304	0.107	8.105	1	0.004	0.738
	Constant	9.424	3.017	9.759	1	0.002	1.238 × 10^4^
**Step 3 ^c^**	putrescine	−2.045	69.121	0.001	1	0.976	0.129
	spermidine	−4.072	139.676	0.001	1	0.977	0.017
	*N*-acetylspermine	−32.047	1.120 × 10^3^	0.001	1	0.977	0.000
	Constant	287.785	9.759 × 10^3^	0.001	1	0.976	9.627× 10^124^
**Step 4 ^d^**	putrescine	−1.517	85.993	0.000	1	0.986	0.219
	spermidine	−2.507	132.834	0.000	1	0.985	0.082
	*N*-acetylspermine	−19.801	1.127× 10^3^	0.000	1	0.986	0.000
	γ-aminobutyric acid	0.415	86.991	0.000	1	0.996	1.514
	Constant	158.206	9.759 × 10^3^	0.000	1	0.987	5.104 × 10^68^

^a^ Variable(s) entered in step 1: putrescine; ^b^ Variable(s) entered in step 2: spermidine; ^c^ Variable(s) entered in step 3: *N*-acetylspermine; ^d^ Variable(s) entered in step 4: γ-aminobutyric acid.

**Table 3:** The relationship of variables in logic regression model for liver cancer plasma and lung cancer plasma.

		Constant	Putrescine	Spermidine	*N*-Acetylspermine	γ-Aminobutyric Acid
Step 1	Constant	1.000	−0.852			
	putrescine	−0.852	1.000			
Step 2	Constant	1.000	−0.878	−0.907		
	putrescine	−0.878	1.000	0.859		
	spermidine	−0.907	0.859	1.000		
Step 3	Constant	1.000	−0.987	−0.962	−0.987	
	putrescine	−0.987	1.000	0.953	0.960	
	spermidine	−0.962	0.953	1.000	0.921	
	*N*-acetylspermine	−0.987	0.960	0.921	1.000	
Step 4	Constant	1.000	−0.308	−0.658	−0.830	−0.440
	putrescine	−0.308	1.000	0.700	0.567	−0.711
	spermidine	−0.658	0.700	1.000	0.559	−0.177
	*N*-acetylspermine	−0.830	0.567	0.559	1.000	0.042
	γ-aminobutyric acid	−0.440	−0.711	−0.177	0.042	1.000

**Table 4:** The variables in the equation of logic regression analysis for liver cancer urine (assigned as 1 as dependent) and lung cancer urine (assigned as 2 as dependent) using the Conditional Method as the analytical method.

		B	S.E.	Wald	df	Sig.	Exp(B)
**Step 1 ^a^**	*S*-adenosyl-l-methionine	0.000	0.000	15.985	1	0.000	1.000
	Constant	−1.784	0.449	15.763	1	0.000	0.168
**Step 2 ^b^**	*N*-acetylspermidine	−0.030	0.007	16.358	1	0.000	0.970
	*S*-adenosyl-l-methionine	0.001	0.000	15.063	1	0.000	1.001
	Constant	−0.395	0.628	0.396	1	0.529	0.674
**Step 3 ^c^**	l-arginine	0.003	0.001	7.878	1	0.065	1.003
	*N*-acetylspermidine	−0.041	0.012	11.197	1	0.001	0.960
	*S*-adenosyl-l-methionine	0.002	0.001	10.233	1	0.001	1.002
	Constant	−2.288	1.142	4.014	1	0.045	0.101

^a^ Variable(s) entered in step 1: *S*-adenosyl-l-methionine. ^b^ Variable(s) entered in step 2: *N*-acetylspermidine. ^c^ Variable(s) entered in step 3: l-arginine.

**Table 5:** The relationship of variables in logic regression model for liver cancer urine and lung cancer urine.

		Constant	*S*-Adenosyl-l-Methionine	*N*-Acetylspermidine	l-Arginine	1,3-Diaminopropane
**Step 1**	Constant	1.000	−0.823			
	*s*-adenosyl-l-methionine	−0.823	1.000			
**Step 2**	Constant	1.000	−0.341	0.103		
	*N*-acetylspermidine	0.103	−0.905	1.000		
	*s*-adenosyl-l-methionine	−0.341	1.000	−0.905		
**Step 3**	Constant	1.000	−0.683	0.566	−0.686	
	l-arginine	−0.686	0.666	−.678	1.000	
	*N*-acetylspermidine	0.566	−0.968	1.000	−0.678	
	*s*-adenosyl-l-methionine	−0.683	1.000	−0.968	0.666	
**Step 4**	Constant	1.000	−0.729	0.378	−0.521	−0.026
	1,3-diaminopropane	−0.026	0.542	−0.903	0.686	1.000
	l-arginine	−0.521	0.698	−0.790	1.000	0.686
	*N*-acetylspermidine	0.378	−0.849	1.000	−0.790	−0.903
	*s*-adenosyl-l-methionine	−0.729	1.000	−0.849	0.698	0.542

## Supplementary Materials

Supplementary materials can be accessed at: http://www.mdpi.com/1420-3049/21/8/1040/s1.

## Abbreviations

The following abbreviations are used in this manuscript:

UHPLC	ultra-high performance liquid chromatography
MS	mass spectrometry
MRM	multiple reaction monitoring
DP	declustering potential
EP	entrance potential
CE	collision energy
CXP	cell exit potential
ESI	electrospray positive ionization
HFBA	heptafluorobutyric acid
ODC	ornithine decarboxylase
DAO	diamine oxidase
SAMDC	*S*-adenosylmethionine decarboxylase
SSAT	spermidine and spermine acetyl transferase
FAD-PAO	flavin adenine dinucleotide-polyamine oxidase
DAP	1,3-diaminopropane
PUT	putrescine
CAD	cadaverine
SPD	spermidine
SPM	spermine
AGM	agmatine
N-PUT	*N*-acetylputrescine
N-SPM	*N*-acetylspermine
N-SPD	*N*-acetylspermidine
L-ORN	l-ornithine
LYS	lysine
L-ARG	l-arginine
SAM	*S*-adenosyl-l-methionine
GABA	γ-aminobutyric acid
