# Supplementary material for: Polyamine Metabolites Profiling for Characterization of Lung and Liver Cancer Using an LC-Tandem MS Method with Multiple Statistical Data Mining Strategies: Discovering Potential Cancer Biomarkers in Human Plasma and Urine
Source: Molecules. 2016 Aug 10;21(8):1040. doi: 10.3390/molecules21081040 (PMC6273014; doi:10.3390/molecules21081040)
Supplement: Supplementary file 1 [file molecules-21-01040-s001.pdf]

Supplementary Materials: Polyamine Metabolites Profiling for Characterization of Lung and Liver Cancer Using an LC-Tandem MS Method with Multiple Statistical Data Mining Strategies: Discovering Potential Cancer Biomarkers in Human Plasma and Urine

Huarong Xu, Ran Liu, Bosai He, Cathy Wenchuan Bi, Kaishun Bi and Qing Li

Table S1. Amounts of polyamine metabolome in plasma (ng/mL) from 50 lung, 50 liver cancer patients and heathy volunteers (mean ± SD).

Analytes	Lung Cancer Patients	Liver Cancer Patients	Heathy Volunteers
DAP	3.74 ± 2.26	5.79 ± 4.54	5.55 ± 4.99
PUT	35.65 ± 16.41 *	77.11 ± 37.14 *	20.35 ± 14.50
CAD	2.58 ± 2.03 **	3.09 ± 2.43 **	21.42 ± 17.49
SPD	5.55 ± 6.79 *	46.24 ± 30.84 **	2.85 ± 2.26
SPM	6.78 ± 3.87	14.24 ± 10.73	12.61 ± 12.02
AGM	71.79 ± 21.97 *	67.08 ± 46.67 *	96.3 ± 54.2
ORN	6374 ± 2429 *	$9.85 \times 10^3 \pm 5.69 \times 10^3$	$9.98 \times 10^3 \pm 10.57 \times 10^3$
LYS	$1.440 \times 10^5 \pm 0.613 \times 10^5$ *	$1.411 \times 10^5 \pm 0.789 \times 10^5$ *	$0.725 \times 10^5 \pm 0.865 \times 10^5$
ARG	$1.042 \times 10^5 \pm 0.468 \times 10^5$ **	$1.005 \times 10^5 \pm 0.636 \times 10^5$ **	$0.232 \times 10^5 \pm 0.262 \times 10^5$
SAM	159.7 ± 90.1 **	131.7 ± 129.0 **	51.86 ± 34.75
NPUT	0.36 ± 0.24	0.52 ± 0.49	0.42 ± 0.36
NSPM	3.12 ± 1.32	7.40 ± 3.39 *	4.64 ± 4.85
NSPD	3.15 ± 0.87	6.21 ± 3.78 *	4.49 ± 5.55
GABA	74.13 ± 32.86 *	91.1 ± 70.5 *	24.14 ± 21.36

* $p < 0.05$, compared to heathy volunteers, ** $p < 0.01$, compared to heathy volunteers.

Table S2. Amounts of polyamine metabolome in urine (ng/mg creatinine) from 50 lung, 50 liver cancer patients and heathy volunteers (mean ± SD).

Analytes	Lung Cancer Patients	Liver Cancer Patients	Heathy Volunteers
DAP	0.98 ± 0.88 *	1.42 ± 2.09 *	4.69 ± 3.91
PUT	19.77 ± 9.13 *	35.12 ± 44.46 *	10.95 ± 11.17
CAD	14.38 ± 16.25 **	26.33 ± 28.77 **	175.8 ± 194.3
SPD	8.88 ± 7.87 *	6.85 ± 8.04 *	2.46 ± 2.41
SPM	90.6 ± 97.7 **	212.5 ± 264.6 **	34.45 ± 40.41
AGM	5544 ± 466 *	5396 ± 4099	3586 ± 3053
ORN	202.1 ± 170.8 *	256.8 ± 305.2 *	149.0 ± 131.8
LYS	$2.844 \times 10^4 \pm 2.537 \times 10^4$ *	$2.878 \times 10^4 \pm 2.303 \times 10^4$ *	$1.115 \times 10^4 \pm 0.601 \times 10^4$
ARG	257.7 ± 281.2 **	699.2 ± 549.6 **	3811 ± 4082
SAM	2283 ± 2074 **	6880 ± 5074 **	$1.002 \times 10^4 \pm 1.635 \times 10^4$
NPUT	1.00 ± 1.09	0.58 ± 0.80 *	2.15 ± 1.55
NSPM	4.44 ± 4.12 *	1.82 ± 1.74 *	8.79 ± 8.04
NSPD	300.1 ± 38.3 **	111.6 ± 128.3	127.3 ± 121.6
GABA	16.07 ± 12.00 **	22.03 ± 16.57 **	169.8 ± 227.9

* $p < 0.05$, compared to heathy volunteers, ** $p < 0.01$, compared to heathy volunteers.
